# Survey on patients’ organisations’ knowledge and position paper on screening for inherited neuromuscular diseases in Europe

**DOI:** 10.1186/s13023-020-01670-8

**Published:** 2021-02-10

**Authors:** F. Lamy, A. Ferlini, ERN EURO-NMD Patient Advisory Board, Dimitrios Athanasiou, Evy Reviers, François Lamy, Jean-Philippe Plançon, Judit Varadine Csapo, Madelon Kroneman, Marisol Montolio, Massimo Marra, Michela Onali, Patrizia Garzena, Teresinha Evangelista

**Affiliations:** 1 Association Française contre les Myopathies, AFM-Téléthon, Evry, France; 2 Unit of Medical Genetics, Department of Medical Sciences, University of Ferrara, Ferrara, Italy; 3 Neuromuscular Morphology Unit, Myology Institute, Groupe Hospitalier Universitaire La Pitié-Salpêtrière, 75013 Paris, France; 4 AP-HP, Centre de Référence des Maladies Neuromusculaires Nord/Est/Ile de France, Sorbonne Université - Inserm UMRS 974, Paris, France

**Keywords:** Neuromuscular diseases, Pre-conception carrier screening, Pre-implantation diagnosis, Prenatal screening, Newborn screening, Patient organisations

## Abstract

**Background:**

The development of new genetic testing methods and the approval of the first treatments raises questions regarding when and how to perform screening for inherited neuromuscular conditions. Screening directives and access to the different techniques is not uniform across Europe. The patient advisory board of the European reference network for rare neuromuscular diseases (NMD) conducted a qualitative study to understand the state of play of screening for inherited NMD in Europe and patients’ needs.

**Results:**

We collected answers from 30 patient organisations (POs) from 18 European countries. Fifteen acknowledge the existence of pre-implantation genetic diagnosis in their country. Regarding prenatal screening, we had 25 positive answers and 5 negative ones. Twenty-four POs mentioned that newborn screening was available in their country. We had some contradictory answers from POs from the same country and in some cases; diseases said to be part of the screening programmes were not hereditary disorders. Twenty-eight organisations were in favour of screening tests. The reasons for the two negative answers were lack of reimbursement and treatment, religious beliefs and eventual insurance constrains. Most POs (21) were in favour of systematic screening with the option to opt-out. Regarding the timing for screening, “at birth”, was the most consensual response. The main priority to perform screening for NMDs was early access to treatment, followed by shorter time to diagnostic, preventive care and genetic counselling.

**Conclusions:**

This is the first study to assess knowledge and needs of POs concerning screening for NMDs. The knowledge of POs regarding screening techniques is quite uneven. This implies that, even in communities highly motivated and knowledgeable of the conditions they advocate for, there is a need for better information. Differences in the responses to the questions “how and when to screen” shows that the screening path depends on the disease and the presence of a disease modifying treatment. The unmet need for screening inherited NMDs should follow an adaptive pathway related to the fast moving medical landscape of NMDs. International coordination leading to a common policy would certainly be a precious asset tending to harmonize the situation amongst European countries.

## Background

In the neuromuscular diseases field the development of new genetic testing methods and the approval of the first treatments that modify the natural history of Mendelian diseases has raised several questions regarding when and how to perform screening for Mendelian conditions.

There several screening techniques that can be used at different time points. Pre-conception carrier screening for severe genetic conditions has been in place for many years, in particular for conditions with a high carrier frequency [1]. A good example is Tay–Sachs disease, which has been the object of population carrier screening in Ashkenazi Jewish descent [2, 3].

Pre-implantation genetic diagnosis (PGD), introduced at the beginning of 1990 is based on in vitro fertilization (IVF) procedures and can in certain circumstances be an additional preventive measure, alternative to postconceptional prenatal diagnosis [4, 5]. PGD can be used when a genetic mutation or structural chromosomal abnormality is already known and detected in the parents.

Prenatal diagnosis allows patients to make informed reproductive decisions and to have genetic counselling about possible foetal outcomes. Recently, non-invasive prenatal testing (NIPT) has entered into routine prenatal testing strategies, and it is already widely offered to detect common chromosomal aneuploidies [6].

Most often, prenatal diagnosis in Mendelian diseases still requires invasive procedures (villus sampling) for foetal-sample collection. Genome-wide single-cell arrays and high-throughput sequencing analyses have increased the ability to detect the genetic anomalies [7, 8].

Newborn screening refers to the early testing of newborns for specific disorders and conditions that if not treated at an early stage can hamper their healthy development. Although at present, newborn screening is applied mostly for inherited metabolic conditions, indeed screening became available for other Mendelian diseases such as Cystic Fibrosis and others. The number of diseases that are screened differs across countries. In European Union countries, newborn screening, like other healthcare policies, is a matter for individual member states [9].

The screening directives and the access to the different techniques mentioned above are not uniform across Europe. An important aspect is a disparity in the knowledge of the general population about the situation in their own country.

The patient advisory board (PAB) of the European reference network (ERN) for rare neuromuscular diseases (EURO-NMD) deemed essential to conduct a qualitative study to take stock of the state of the art regarding screening for inherited neuromuscular disorders (NMD) in Europe from a patient point of view. With that aim, a questionnaire with four main points [(1) Does screening exist in your country? (2) Is screening done systematically? (3) Who supports its costs? and (4) What are your organisation’s views on screening?] was sent to 115 patient organisations (POs).

## Methods

The PAB of the ERN EURO-NMD developed an online questionnaire aiming at getting a qualitative insight on the degree of knowledge patient associations have about their National screening system and to try to understand their needs regarding screening for genetic/inherited neuromuscular diseases (Additional file 1: Supplementary Material 1—questionnaire). To avoid misinterpretations, we attached a glossary of terms to the survey (Table 1).

**Table 1:** Glossary of terms

Glossary of terms	
Pre-conception carrier screening	Allows determining whether a couple is at risk of conceiving a child with a genetic disorder
Pre-implantation diagnosis	Is the genetic profiling of embryos prior to implantation in case of In Vitro Fertilization (IVF) procedures
Prenatal screening	Takes place at the early stages of pregnancy and aims to detect whether a foetus is affected by a list of conditions
Newborn screening	Takes place shortly after birth and aims to detect if an infant is affected by a list of conditions

The questionnaire was sent to 115 European POs using two email campaigns (June and July 2019). The survey stayed open from June to October 2019. The answers were anonymised.

## Results

### Demographics

**Fig. 1:**
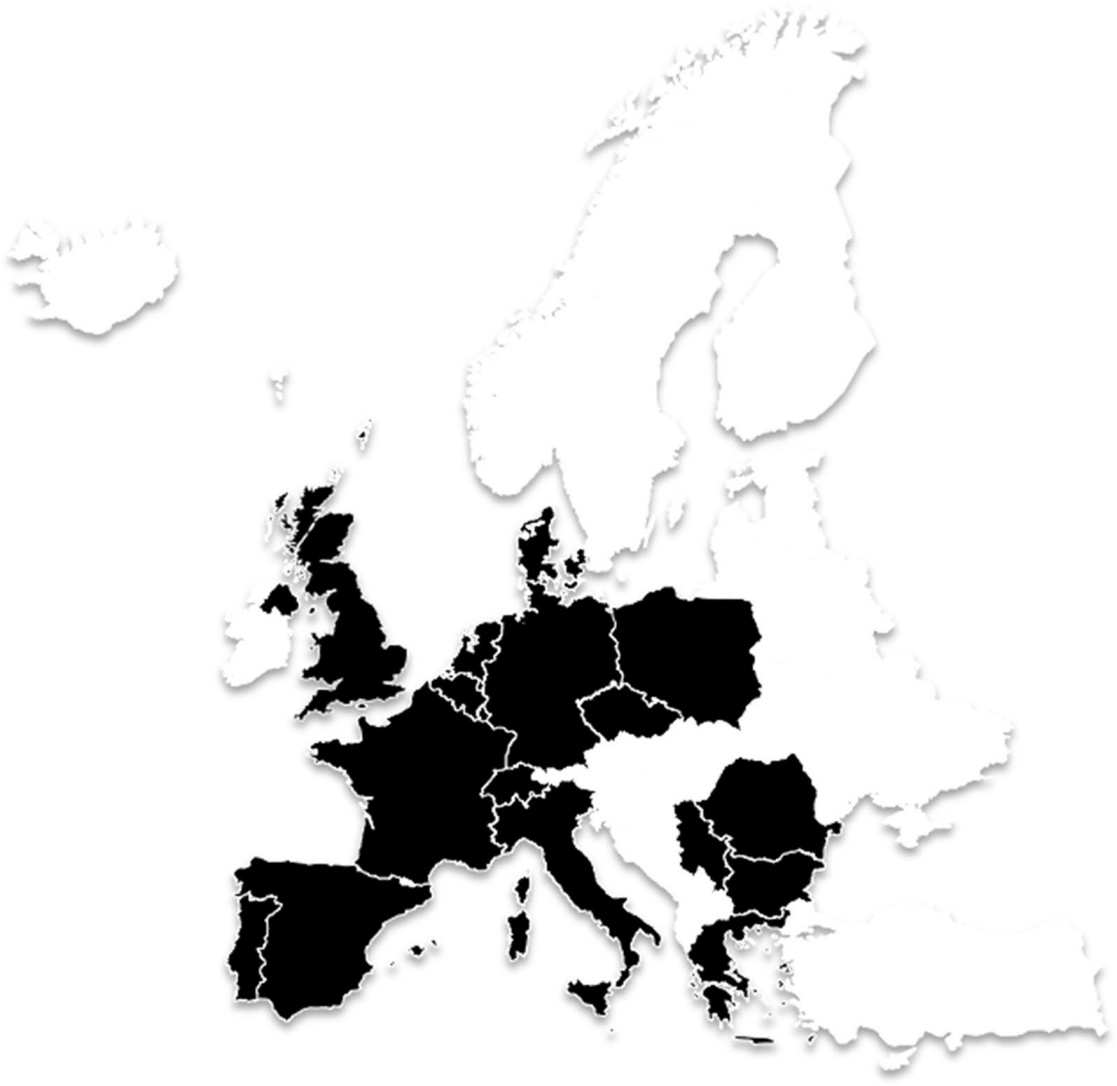
Legend—Number of countries (18) and the number of answers per country: Belgium (1), Bulgaria (1), Czech Republic (1), Denmark (1), Germany (1), Greece (1), Italy (1), Luxembourg (1), The Netherlands (1), Poland (1), Portugal (1), Serbia (1), Switzerland (1), United-Kingdom (1), France (2), North Macedonia (2), Romania (2), Spain (10)

We collected answers from 30 POs (response rate of 26%) based in 18 European countries. Of these 18 countries, 17 were EU countries, and 10 were members of the ERN EURO-NMD. (Fig. 1).

**Fig. 2:**
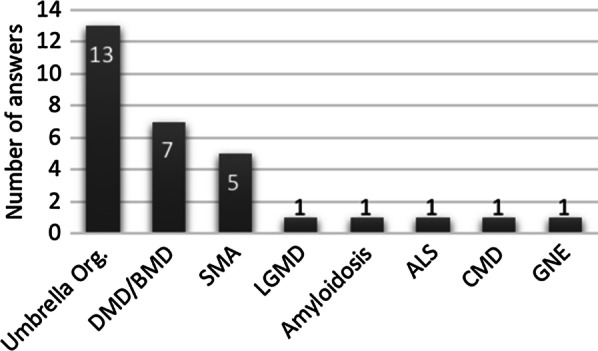
Legend: Number of responses per type of patient organisation. Umbrella Org.—“all-neuromuscular diseases organisations”; *DMD/BMD* Duchenne and Becker muscular dystrophy organisations, *SMA* spinal muscular atrophy organisations, *LGMD* limb girdle muscular dystrophies organisation, *CMD* congenital muscular dystrophies organisations, *GNE* GNE myopathy organisation (GNEM)

Two main categories of patients’ associations were included in the survey: “all-neuromuscular diseases organisations” that represent all NMDs and those dedicated to a disease or thematic group of NMD. The “all-neuromuscular diseases organisations” provided 43.3% (13/30) of the answers, and the disease-specific organisations 56.7%. Figure 2, represents the disease coverage across organisations. It is noticeable that besides the “all-neuromuscular diseases organisations”, the majority were organisations dedicated to Duchenne/Becker muscular dystrophy (DMD/BMD) or spinal muscular atrophy (SMA).

### The current reported situation regarding screening in the different countries

#### Pre-implantation diagnostic (PGD)

Regarding the question “Is PGD in place in your country?” 15/30 associations from 10/18 countries (Spain, Romania, Portugal, The Netherlands, Greece, Germany, France, Bulgaria, Belgium, UK) responded affirmatively. When we analysed the answers per country, we noticed an inconsistency. Half of the associations from Spain (5/10) and Romani (1/2) mentioned that PGD was available, while the other 50% stated that PDG was not available.

When PGD was available, we specifically asked if and which NMDs were included. For 14/15, the answer was positive; only for Portugal, it was stated that PGD did not include NMD. According to the responses collected and depending on the country, PGD is available for Duchenne and Becker muscular dystrophy (DMD), spinal muscular atrophy (SMA), Charcot-Marie-Tooth neuropathy (CMT), amyotrophic lateral sclerosis (ALS), myasthenia gravis (MG), myotonic dystrophy (MD) and facioscapulohumeral muscular dystrophy (FSHD). There is an obvious caveat in the answers we have received as some non-genetic diseases were mentioned (Table 2).

**Table 2:** Availability of screening methods for neuromuscular diseases

Country	Type of organisation	Is PGD available?	Includes NMD?	Is PNS in place?	Includes NMD?	Is NBS in place?	Includes NMD?
BE	Disease specific	Yes	Yes	Yes	Yes	Yes	Yes
BG	All NMD	Yes	Yes	Yes	Yes	Yes	Yes
CZ	Disease specific	No	No	Yes	No	Yes	No
DK	All NMD	No	No	Yes	No	Yes	No
FR	Disease specific	Yes	Yes	Yes	Yes	No	No
FR	All NMD	Yes	Yes	Yes	Yes	Yes	No
DE	All NMD	Yes	Yes	Yes	Yes	Yes	No
GR	All NMD	Yes	Yes	Yes	Yes	Yes	Yes
IT	Disease specific	No	No	Yes	Yes	Yes	Yes
LU	All NMD	No	No	Yes	No	Yes	No
NL	All NMD	Yes	Yes	No	No	Yes	No
MK	Disease specific	No	No	Yes	Yes	No	No
MK	All NMD	No	No	Yes	Yes	No	No
PL	All NMD	No	No	Yes	Yes	Yes	No
PT	All NMD	Yes	No	No	No	Yes	No
RO	Disease specific	No	No	Yes	No	Yes	No
RO	Disease specific	Yes	Yes	Yes	Yes	Yes	Yes
RS	Disease specific	No	No	Yes	No	No	No
ES	All NMD	No	No	No	No	No	No
ES	Disease specific	No	No	Yes	No	Yes	No
ES	Disease specific	No	No	Yes	No	Yes	No
ES	All NMD	Yes	Yes	Yes	Yes	Yes	Yes
ES	Disease specific	Yes	Yes	No	No	Yes	Yes
ES	Disease specific	Yes	Yes	Yes	Yes	Yes	Yes
ES	All NMD	Yes	Yes	Yes	Yes	Yes	Yes
ES	Disease specific	No	No	Yes	No	No	No
ES	Disease specific	Yes	Yes	Yes	Yes	Yes	Yes
ES	Disease specific	No	No	No	No	Yes	No
CH	Disease specific	No	No	No	No	Yes	No
UK	Disease specific	Yes	Yes	Yes	No	Yes	No

Representation of the answers across countries and type of PO regarding the different screening techniques

#### Prenatal screening (PNS)

Regarding the availability of prenatal screening (PNS) in different countries, we had 25 positive answers and 5 negative ones corresponding to 3 countries: Spain, Portugal and Switzerland.

Once more, we had collected some conflicting answers with 7 Spanish associations saying that PNS was available and three saying that it was not. Some of the diseases included in the responses were diseases for which PNS is not available. PNS would cover, depending on the countries, Duchenne and Becker muscular dystrophy (DMD), spinal muscular atrophy (SMA), Charcot-Marie-Tooth neuropathy (CMT), motor neuron disease (ALS), myasthenia gravis (MG), myotonic dystrophy (DM), Limb-Girdle Muscular dystrophy (LGMD) and Amyloidosis (AM) (Table 2).

#### Newborn screening (NBS)

To the question “is newborn screening available in your country”, 24 associations said yes and 6, from 4 different countries, said no (France—1, North Macedonia—2, Serbia—1, Spain—2). Fourteen associations mentioned that NBS did not include NMD and ten said that NMD was part of the newborn screening program in their country (Belgium (1), Bulgaria (1), Greece (1), Italy (1), Romania (1), Spain (5)). In Bulgaria, all genetically transmitted NMD are reported as being part of the NBS program. NMD that were referred to as being part of the NBS programs were DMD, SMA, POMPE (Italy), Myotonic dystrophy (Spain). NBS was recently approved in the Netherlands for SMA and at it had been refused for Pompe disease. In Italy, in December 2018, an amendment was adopted which extended the newborn screening to NMD although the diseases to be included were still undefined. In June 2019 and for two years, a pilot project for NBS for SMA was launched in the regions of Lazio and Tuscany. In addition, Germany has in place a pilot project for NBS for SMA.

Once more, we noticed a lack of information regarding the real situation of NBS in each country. All European countries, except Albania, have NBS programs, the main differences being the number of diseases included, between 1 and 30. Therefore, NBS is available in almost all European countries, and the disease coverage was extended after the introduction of tandem mass spectrometry.

### Views of patients’ organisations regarding screening for NMD

As screening for genetic conditions can conjure very personal believes, either religious or ethical and raise economic issues we wanted to know if the patients’ organisations (POs) involved in this survey were in favour of screening for the conditions that are relevant to their organisation. Twenty-eight organisations were in favour of testing for the conditions they advocate for. The reasons given for the two negative answers were lack of reimbursement, lack of treatment, religious beliefs and that a positive test would make it difficult or very expensive to get a mortgage or an insurance (Table 3).

**Table 3:** Reasons why the POs were not in favour of screening

Country	Absence of disease-modifying treatment	Personal, cultural or religious beliefs	Lack of reimbursement	Pricy/ impossible mortgage/insurance
Poland	1	3	1	1
Spain	2	1	1	5

Answers ranged from 1 to 5. 1 being the most important reason, 5 the least

From the POs in favour of screening, we further wanted to know when and how they preconized it should be implemented, if it should be systematic and if it was preferable to implement an opt-in or opt-out system. A summary of the answers can be seen in Table 4.

**Table 4:** How and when to screen

Modality of screening	Number of answers	When should screening take place?	Number of answers
Systematic with the option to opt-out	21	At birth	9
		Early pregnancy	6
		Preconception	4
		Depends on the disease and the situation	3
Systematic	4	At birth	2
		Early pregnancy	1
		Preconception	1
Not systematic with the possibility to opt-in	3	At birth	1
		Depends on the disease and the situation	1
		Preconception	1

Most PO (21) were in favour of systematic screening with the option to opt-out. The opt-out option was preferred regardless of the type of association, disease-specific or all-neuromuscular diseases (Table 5).

**Table 5:** How to screen: answers from POs by the pathologies they represented

	DMD	SMA	NMD	OTHER	TOTAL
Systematic with the option to opt-out	5 (83%)	5 (100%)	7 (58%)	4 (80%)	21
Systematic	1 (17%)	0	3 (25%)	0	4
Not systematic with the possibility to opt-in	0	0	2 (17%)	1 (20%)	3

“DMD” stands for DMD/BMD patients’ organisations (n = 6), “SMA” for SMA POs (n = 5), “NMD” for all neuromuscular diseases POs (n = 12), “Other” for other neuromuscular diseases specific POs (n = 5)

When we look at the answers for when should screening take place, we see divided opinions; however, “at birth” seem to reunite most of the responses (12).

When we look at the organisations devoted to SMA, it is unanimous that the screening should occur at birth. However, early pregnancy and pre-conception screening were also strongly envisaged for other neuromuscular diseases organisations. (Table 6).

**Table 6:** When to screen answers from POs by the pathologies they represented

When	DMD	SMA	NMD	OTHER	TOTAL
Pre-conception	3 (50%)	0	2 (17%)	1 (20%)	6
Early pregnancy	2 (33%)	0	2 (17%)	3 (60%)	7
At birth	1 (17%)	5 (100%)	5 (42%)	1 (20%)	12
It depends	0	0	3 (25%)	0	3

“DMD” stands for DMD/BMD patients’ organisations (n = 6), “SMA” for SMA POs (n = 5), “NMD” for all neuromuscular diseases POs (n = 12), “Other” for other neuromuscular diseases specific POs (n = 5)

We also inquired about the reason(s) why screening should take place and asked the participants to select the relevant reasons and rank them (1 being the most important, 6 the least). Summary of the answers can be seen in Table 7.

**Table 7:** Reasons why POs where in favour of screening

Rank	Shorter time to diagnostic	Early access to treatments	Inclusion in clinical trials	Preventive care	Genetic counselling
1	13 (50%)	18 (69%)	8 (33%)	11 (44%)	13 (48%)
2	7 (27%)	5 (19%)	6 (25%)	9 (36%)	7 (26%)
3	3 (12%)	1 (4%)	4 (17%)	3 (12%)	4 (15%)
4	2 (8%)	2 (8%)	0	2 (8%)	2 (7%)
5	1 (4%)	0	5 (21%)	0	1 (4%)
6	0	0	1 (4%)	0	0

Aggregated results for all patients’ organisations (n = 28). 1 = most important, 6 = least important

When we analysed all the answers (n = 28) priority was given to early access to treatment, followed with equal importance by shorter time to diagnosis, preventive care and genetic counselling. The inclusion in clinical trials was the question that scored the lowest value in terms of importance. The answers of the “all-neuromuscular diseases organisations” were very similar to the aggregated responses.

However, when we analyse at the specific pathology level answers can be radically different.

On the one hand, for the SMA organisations, priority was given to early access to treatment. On the other hand, for DMD associations, priority was given to preventive care and genetic counselling.

## Discussion

Advances in the treatment of NMDs has brought to the limelight the need for an accurate genetic diagnosis, early in the disease process, to allow treatments to be most effective.

This is the first study to specifically assess the knowledge and the needs of NMD POs concerning screening methods. We included responses from POs from 18 countries (17 are part of the EU) with ethnically and genetically heterogeneous populations and different economic backgrounds.

The knowledge of the individual POs regarding the availability of screening methods in the different countries is quite uneven, independently of being “all-NMD” or disease-specific organisations. This implies that, even in communities highly motivated and knowledgeable of the conditions they advocate for, there is a need for better information. The different European countries, through their health services, should make available in lay language accurate information regarding the different screening techniques available in their health systems.

According to the report from the JRC from 2007 on “Preimplantation Genetic Diagnosis in Europe” [10] PGD is well established in Europe and provided in many European countries. Regulations, practices, professional standards and accreditation requirements are markedly different between the Member States. According to this report, 21 European countries can offer PDG and/or IVF with an equal distribution between private and public centres. With the PGD only centres concentrating in the private sector (78%).

More than half of the countries according to the POs in our survey have PGD in place. The fact that we got conflicting responses from POs in the same country prompted us to compare our answers with the ones given by professionals in the JRC survey. The countries that were part of the JRC survey did not overlap exactly with the ones in our survey, and this made data comparison slightly difficult. Countries in our survey that were not present in the JRC survey were Bulgaria, Luxembourg, Poland, Serbia, North Macedonia, and Romania. On the other hand, we were not able to collect answer from POs from Austria, Cyprus, Finland, Hungary, Lithuania, Slovakia, Sweden and Turkey. The conflicting responses came from Spain and Romania; and in fact, Spain is one of the countries with more centres in Europe offering this service and one of the biggest cross border providers. In Romania, PGD is still quite inaccessible due to the high costs and lack of reimbursement through the public health system. [11]. POs in Czech Republic, Denmark, Italy, Luxembourg, North Macedonia, Poland, Serbia and Switzerland are unaware of the possibility of PGD in their country however it is available in, the Czech Republic, Denmark, Italy and Switzerland [10, 12, 13].

The discrepancies we have observed in the answers might also be due to the modalities (public or private services) of PGD offer in each country. Indeed, it is conceivable that knowledge about the existence of the private laboratories might not be so accurate; explaining in part the inconsistent in the data.

Prenatal screening is, according to our survey, well disseminated across the different countries. Once more, we got a discordant answer from centres in Spain that may reflect a lack of awareness and means that there is a need for education and good dissemination of information. For Portugal and Switzerland POs stated that prenatal screening is not available. Portugal has in place since July 1997 an official text concerning organisation and procedures of prenatal diagnosis (PND) at the national level [14]. In addition, Switzerland has a PND policy in place for foetal malformations [15]. There is a lack of information in the literature regarding the present policies and offers of PND across Europe. The NMDs most frequently referred to as being covered were DMD, SMA, CMT, ALS, MG, DM, LGMD and AM. The authors believe that, once more, these answers reflect a lack of information regarding the techniques and the diagnostic possibilities of PND.

In Europe, each country develops and is responsible for its health care policy, including on newborn screening. According to the report on the practices of newborn screening for rare disorders (RP-NBS) from 2016 [16] the vast majority of European countries have laws or regulations mandating newborn screening, however in only a few of these countries there is an obligation to participate to the NBS plan. All European countries, except Albania, have NBS programs, the main differences being the number of diseases included, between 1 and 30. Most of the diseases screened are metabolic disorders (that includes some NMD), endocrinological and haematological conditions and cystic fibrosis. In our survey, POs from 4 countries (France, Serbia, North Macedonia and Switzerland) were not aware of the existence of an NBS program in their country.

POs mentioned that the same NMD, as in the PNS question, were included in the NBS programs. However, the current techniques cannot detect some of them. Additionally, most countries still base their decision to add a disease to an NBS panel in the criteria by Wilson and Jungner from 1968 [17]. These criteria are based mostly on the fact that the disorder would benefit from earlier intervention. The approval of treatments for POMPE Disease, SMA and DMD have launched the debate across the different stakeholders regarding the need to include these diseases in the NBS programs. Historically NBS for DMD started in 1975 in the USA with the measurement of creatinine kinase on newborn male blood spots. More than ten programs were implemented over time around the globe (Wales, France, Australia, China, New Zealand, Cyprus, Belgium, Germany, Canada, Scotland, and the United States) [18]. However, as of today none of them led to the introduction of DMD to any national screening program. The first pilot program for Pompe disease began in 2005 in Taiwan. Currently, NBS for POMPE is available in Italy [19] and was refused in The Netherlands in 2015 because it was not possible to distinguish between the infantile and adult forms of Pompe, which would not be eligible according to the Wilson et al. criteria. The inclusion could be reconsidered if this distinction become available. With the approval of nusinersen in late 2016, spinal muscular atrophy is being considered for NBS across the world [20] and several pilots are running in Europe (Southern Belgium, Germany and Italy) [21, 22]. This situation raises serious issues since to achieve therapeutic benefit infants need to be screened and treated soon after birth (according to the concept of therapeutic temporal window) and on the other hand, most countries face technical and economic difficulties to implement NBS. The delay between drug approval and NBS implementation dramatically impairs patients’ conditions with very diverse outcomes from one country to another.

Health policies must acknowledge the idiosyncratic nature and varied aetiology of rare diseases, meet the needs of people diagnosed with rare diseases, take into account it rapidly moving landscape and aim to improve management and reduce the associated human, community and system cost. To achieve these goals is essential to take in consideration the Patients views regarding the different policies. In our study, we were interested in assessing the views of POs regarding screening for genetic, inherited NMDs. Independently of the ethnic and cultural heterogeneity, most POs (28 out of 30) were in favour of screening for the disease (s) relevant for the organisation irrespectively of the existence of a disease-modifying medical treatment. If we consider that only few mitochondrial diseases and more recently SMA are included in national screening programs this study reveals that screening NMD is a largely unmet need. It shows as well that Wilson and Jungner criteria should evolve to take into account today’s rare disease landscape [23].

The motives against screening raised by two POs were cultural and religious beliefs, together with economic problems regarding insurance companies. The legislators must be able to ensure the confidentiality of the tests and should adapt the directives to the religious and cultural background of the populations. For most POs screening should be systematic with a possibility to refuse giving space for individual decision. According to POs screening should be done at birth or early in the pregnancy. The primary motivations for screening were early access to treatment followed by shorter time to diagnosis. Not surprisingly in a disease such as SMA, where there is a treatment, the consensus was to have the screening done at birth, and the main goal was early access to treatment. For DMD, the responses were more divided with the majority considering preconception screening or early pregnancy screening stressing the need for disease prevention and genetic counselling.

In conclusion, most POs are in favour of screening, preconception, early during pregnancy or as part of NBS. The motivations seem different when we are in the presence of a disease with or without a treatment. When there is no treatment, the aim is mostly the prevention of the disease via family planning. It is also interesting to note that “a shorter time to diagnosis” is a fundamental goal of the PO so that they can break the vicious circle of delayed diagnosis and associated consequences.

The responses obtained to the questions “how to screen” and “when to screen” show that decisions regarding the diseases to be included in screening programmes depends mostly on the presence of a disease modifying treatment and on the need to reduce diagnostic delay.

The screening programmes should be flexible enough to adjust to the present fast moving landscape of treatments for NMDs. This need for flexibility is not complacent with the current status quo that implies a long and heavy process to add a disease to national screening programs.

International coordination in the domain leading to common policies would certainly be a precious asset tending to harmonize the situation from one country to another and speeding up the process of adding a disease to national screening programs. This international coordination should also anticipate market authorisation to avoid that the absence of screening program prevents drug access with dramatic consequences for the patients.

Besides, recent initiatives in the domain of Artificial Intelligence or signalling instrument could contribute to cost effective solutions. Without replacing standard biological and/or genetic diagnostic tests, it could be used prior to them to assess a risk. A signalling instrument was developed in the Netherlands that detects 80% of 12–36 months old boys with Duchenne Muscular Dystrophy [24].

The further important consequence of early diagnosis through newborn screening is allowing family planning of reproductive choices and disease prevention, which certainly impact on quality of life of patients and families.

### Strengths and limitations

A key strength of this work is its coverage of a large number of European countries with different societal backgrounds. It also states the direct opinion of PO without any filter by clinicians and is, to our knowledge and after a literature review, the first work that tries to collect patient reported data regarding screening for inherited neuromuscular diseases in Europe. The fact that we have collect a large number of answers from Spain when compared to other countries can be seen as a bias but indeed it allowed to determine how uneven is the knowledge in one country. This aspect was substantiated every time we were able to collect answers from more than one PO in the same country. Nevertheless, it is important to consider the limitations of this work when interpreting its findings. While the survey was sent to 115 PO, we have received a limited number of answers (26% of responders) and we were only able to cover 18 European countries. Though low responder percentages are common in healthcare surveys, we accept this hinders the representativeness of this study.

We will, on a second step, try to extend our survey and collect more data in each country. This will allow to better understand if the disparity found in the Spanish PO is also present in other countries.

## Conclusions

This survey shows the need to develop better information tools for laypeople so that patients and their families have easy access to information about the availability and the techniques used for screening in different European countries. This information is also essential to facilitate cross-border access to these interventions, and this way reduce the inequality of access in the different Member States.

Screening for genetic/inherited NMDs is a priority for the POs; it will enable them to have early access to treatments, to promote disease prevention and to reduce the time to diagnostic. Unlike what we expected, cultural and ethical beliefs, although important, did not show in this survey as impeditive for screening.

The largely unmet need for screening genetic/inherited NMDs should follow an adaptive pathway related to the fast moving medical landscape of NMDs. International coordination in the domain leading to common policy would certainly be a precious asset tending to harmonize the situation from one country to another. IT/AI solutions could offer a cost effective flexible solution facilitating screening implementation process.

## Supplementary Information

**Additional file 1.** Questionnaire Sent to the patients.

## Data Availability

Data sharing is not applicable to this article as no datasets were generated or analysed during the current study.
