# Supplementary material for: Survey on patients’ organisations’ knowledge and position paper on screening for inherited neuromuscular diseases in Europe
Source: Orphanet J Rare Dis. 2021 Feb 10;16:75. doi: 10.1186/s13023-020-01670-8 (PMC7874448; doi:10.1186/s13023-020-01670-8)
Supplement: Supplementary file 1 — Additional file 1. Questionnaire Sent to the patients. [file 13023_2020_1670_MOESM1_ESM.docx]

Additional file 1 : “Questionnaire Sent to the patients”

| **1.** | **Who is completing this questionnaire?** | | | |
| --- | --- | --- | --- | --- |
|  |  |  | | |
| 1.1 | Patient organization name |  | | |
|  |  | | | |
| 1.2 | Country |  | | |
|  |  | | | |
| 1.3 | What are the hereditary neuromuscular conditions which are relevant your organisation? | | | |
| 🞎 | All neuromuscular diseases | | | |
| 🞎 | Spinal muscular atrophy (SMA) | | | |
| 🞎 | Duchenne muscular dystrophy | | | |
| 🞎 | Becker muscular dystrophy | | | |
| 🞎 | Steinert/ Myotonic dystrophy | | | |
| 🞎 | Limb girdle myopathy Limb-girdle muscular dystrophy | | | |
| 🞎 | Pompe disease | | | |
| 🞎 | Myasthenia gravis | | | |
| 🞎 | Charcot-Marie-Tooth | | | |
| 🞎 | Amyloidosis | | | |
| 🞎 | FSHD | | | |
| 🞎 | Amyotrophic Lateral Sclerosis | | | |
| 🞎 | Other. Which? | | | |
|  |  | | | |
| **2.** | **Current situation regarding screening in your country** | | | |
|  |  | | | |
| 2.1 | Is **pre-implantation diagnostis** in place in your country? | | 🞎 yes | 🞎 no |
|  |  | | | |
| 2.1.1 | If yes, does it include neuromuscular diseases? | | 🞎 yes | 🞎 no |
|  |  | | | |
| 2.1.2 | If yes, which hereditary neuromuscular diseases? | | | |
| 🞎 | All neuromuscular diseases | | | |
| 🞎 | Spinal muscular atrophy (SMA) | | | |
| 🞎 | Duchenne muscular dystrophy | | | |
| 🞎 | Becker muscular dystrophy | | | |
| 🞎 | Steinert /Myotonic dystrophy | | | |
| 🞎 | Limb girdle myopathy Limb-girdle muscular dystrophy | | | |
| 🞎 | Pompe disease | | | |
| 🞎 | Myasthenia gravis | | | |
| 🞎 | Charcot-Marie-Tooth | | | |
| 🞎 | Amyloidosis | | | |
| 🞎 | FSHD | | | |
| 🞎 | Amyotrophic Lateral Sclerosis | | | |
| 🞎 | Other. Which? | | | |
|  |  | | | |
| 2.1.3 | If yes, how is it offered? | | | |
| 🞎 | Systematically | | | |
| 🞎 | Systematically proposed, with the possibility to refuse it | | | |
| 🞎 | Not proposed systematically, with the possibility to ask for it | | | |
| 🞎 | Proposed when an hereditary disease is present in the family | | | |
| 🞎 | Other | | | |
|  |  | | | |
| 2.1.4 | If yes, how is it financed? | | | |
| ¨ | Systematically | | | |
| 🞎 | Systematically proposed, with the possibility to refuse it | | | |
| 🞎 | Not proposed systematically, with the possibility to ask for it | | | |
| 🞎 | Proposed when an hereditary disease is present in the family | | | |
| 🞎 | Other | | | |
|  |  | | | |
| 2.2 | Is **pre natal screening** in place in your country? | | 🞎 yes | 🞎 no |
|  |  | | | |
| 2.2.1 | If yes, does it include neuromuscular diseases? | | 🞎 yes | 🞎 no |
|  |  | | | |
| 2.2.2 | If yes, which hereditary neuromuscular diseases? | | 🞎 yes | 🞎 no |
| 🞎 | All neuromuscular diseases | | | |
| 🞎 | Spinal muscular atrophy (SMA) | | | |
| 🞎 | Duchenne muscular dystrophy | | | |
| 🞎 | Becker muscular dystrophy | | | |
| 🞎 | Steinert/ Myotonic dystrophy | | | |
| 🞎 | Limb girdle myopathy Limb-girdle muscular dystrophy | | | |
| 🞎 | Pompe disease | | | |
| 🞎 | Myasthenia gravis | | | |
| 🞎 | Charcot-Marie-Tooth | | | |
| 🞎 | Amyloidosis | | | |
| 🞎 | FSHD | | | |
| 🞎 | Amyotrophic Lateral Sclerosis | | | |
| 🞎 | Other. Which? | | | |
|  |  | | | |
| 2.2.3 | If yes, how is it offered? | | | |
| 🞎 | Systematically | | | |
| 🞎 | Systematically proposed, with the possibility to refuse it | | | |
| 🞎 | Not proposed systematically, with the possibility to ask for it | | | |
| 🞎 | Proposed when an hereditary disease is present in the family | | | |
| 🞎 | Other | | | |
|  |  | | | |
| 2.2.4 | If yes, how is it financed? | | | |
| 🞎 | By national health care system | | | |
| 🞎 | By private health insurance | | | |
| 🞎 | By people’s money | | | |
| 🞎 | Other | | | |
|  |  | | | |
| 2.3 | Is **new born screening** in place in your country? | | 🞎 yes | 🞎 no |
|  |  | | | |
| 2.3.1 | If yes, does it include hereditary neuromuscular diseases? | | 🞎 yes | 🞎 no |
|  |  | | | |
| 2.3.2 | If yes, which neuromuscular diseases? | | | |
| 🞎 | All neuromuscular diseases | | | |
| 🞎 | Spinal muscular atrophy (SMA) | | | |
| 🞎 | Duchenne muscular dystrophy | | | |
| 🞎 | Becker muscular dystrophy | | | |
| 🞎 | Steinert/ Myotonic dystrophy | | | |
| 🞎 | Limb girdle myopathy Limb-girdle muscular dystrophy | | | |
| 🞎 | Pompe disease | | | |
| 🞎 | Myasthenia gravis | | | |
| 🞎 | Charcot-Marie-Tooth | | | |
| 🞎 | Amyloidosis | | | |
| 🞎 | FSHD | | | |
| 🞎 | Amyotrophic Lateral Sclerosis | | | |
| 🞎 | Other. Which? | | | |
|  |  | | | |
| 2.3.3 | If yes, how is it offered? | | | |
| ¨ | Systematically | | | |
| 🞎 | Systematically proposed, with the possibility to refuse it | | | |
| 🞎 | Not proposed systematically, with the possibility to ask for it | | | |
| 🞎 | Proposed when an hereditary disease is present in the family | | | |
| 🞎 | Other | | | |
|  |  | | | |
| 2.3.4 | If yes, how is it financed? | | | |
| 🞎 | By national health care system | | | |
| 🞎 | By private health insurance | | | |
| 🞎 | By people’s money | | | |
| 🞎 | Other | | | |
|  |  | | | |
| 2.4 | Please add anything you would like to share about current screening in your country | | | |
|  | | | | |
| **3.** | **Your opinion on screening in your country** | | | |
|  |  | | | |
| 3.1 | Are you in favour of systematic screening of the condition(s) which is relevant your organisation? | | 🞎 yes | 🞎 no |
|  |  | | | |
| 3.1.1 | If yes, should it be? | | | |
| 🞎 | Systematic | | | |
| 🞎 | Systematically proposed, with the possibility to refuse it | | | |
| 🞎 | Not proposed systematically, with the possibility to ask for it | | | |
|  |  | | | |
| 3.1.2 | If yes, at which point (several answers possible)? | | | |
| 🞎 | Pre-conception | | | |
| 🞎 | Pre-implantation | | | |
| 🞎 | Early pregnancy | | | |
| 🞎 | At birth | | | |
| 🞎 | Other. Which moment ? | | | |
|  |  | | | |
| 3.1.3 | If yes, for which reasons (several answers possible, please sort them)? | | | |
| 🞎 | Avoid lengthy diagnostic process | | | |
| 🞎 | Possibility for early access to treatments | | | |
| 🞎 | Possibility for inclusion in clinical trials | | | |
| 🞎 | Possibility for preventive care (physio, cognitive, …) | | | |
| 🞎 | Parents’ access to genetic counseling (in case of future pregnancy) | | | |
| 🞎 | Other. Which reasons ? | | | |
|  |  | | | |
| 3.1.4 | If no, for which reasons(several answers possible, please sort them)? | | | |
| 🞎 | Absence of disease modifying treatment available when diagnosed | | | |
| 🞎 | Personal/cultural/religious | | | |
| 🞎 | No reimbursement | | | |
| 🞎 | Would make pricy/impossible to get a mortgage/insurance | | | |
| 🞎 | Other. Which reasons ? | | | |
|  |  | | | |
| 3.2 | Please add anything you would like to share about your views on screening | | | |
|  | | | | |
